# Foundation models in plant molecular biology: advances, challenges, and future directions

**DOI:** 10.3389/fpls.2025.1611992

**Published:** 2025-06-03

**Authors:** Feng Xu, Tianhao Wu, Qian Cheng, Xiangfeng Wang, Jun Yan

**Affiliations:** Frontiers Science Center for Molecular Design Breeding, State Key Laboratory of Maize Bio-breeding, National Maize Improvement Center, College of Agronomy and Biotechnology, China Agricultural University, Beijing, China

**Keywords:** foundation model, plant, molecular biology, large language model, transformer

## Abstract

A foundation model (FM) is a neural network trained on large-scale data using unsupervised or self-supervised learning, capable of adapting to a wide range of downstream tasks. This review provides a comprehensive overview of FMs in plant molecular biology, emphasizing recent advances and future directions. It begins by tracing the evolution of biological FMs across the DNA, RNA, protein, and single-cell levels, from tools inspired by natural language processing (NLP) to transformative models for decoding complex biological sequences. The review then focuses on plant-specific FMs such as GPN, AgroNT, PDLLMs, PlantCaduceus, and PlantRNA-FM, which address challenges that are widespread among plant genomes, including polyploidy, high repetitive sequence content, and environment-responsive regulatory elements, alongside universal FMs like GENERator and Evo 2, which leverage extensive cross-species training data for sequence design and prediction of mutation effects. Key opportunities and challenges in plant molecular biology FM development are further outlined, such as data heterogeneity, biologically informed architectures, cross-species generalization, and computational efficiency. Future research should prioritize improvements in model generalization, multi-modal data integration, and computational optimization to overcome existing limitations and unlock the potential of FMs in plant science. This review serves as an essential resource for plant molecular biologists and offers a clear snapshot of the current state and future potential of FMs in the field.

## Introduction

In recent years, large language models (LLMs) based on Transformer architecture, such as BERT and GPT, have revolutionized NLP (Vaswani et al., 2017; Radford et al., 2018; Devlin et al., 2019). LLMs use self-supervised learning (SSL) to learn semantic patterns and contextual relationships from massive text datasets (Hou et al., 2024). Many employ a two-stage pre-training and fine-tuning process, which enables remarkable generalization, often eliminating or substantially reducing the need for the task-specific feature engineering typical of traditional machine learning approaches (Hou et al., 2024). The self-attention mechanism in LLMs efficiently captures long-range dependencies in sequential data, supported by increased training speed through parallel computing (Hou et al., 2024). Recently, LLMs have extended beyond NLP into scientific domains reliant on sequential data, such as biology, where they offer a new computational framework for modeling biological sequences, inferring structure–function relationships, and predicting the effects of genetic variation (Liu et al., 2024; Zhang et al., 2025).

The sequences of biological macromolecules, including DNA, RNA, and proteins, exhibit a hierarchical structure analogous to natural language, making LLMs well-suited to advance biological foundation models (FMs) (Liu et al., 2024). Most biological FMs are built on LLMs due to their ability to effectively model the hierarchical and context-dependent relationships in biological sequences. Although alternative approaches exist, LLMs have become the dominant framework for biological FMs because of their exceptional performance in capturing complex patterns in sequential data. However, most existing biological FMs are trained on human or animal data, limiting their application in plant sciences. Plant genomes often pose specialized challenges, including polyploidy (*e.g.*, hexaploid wheat) (Walkowiak et al., 2020), extensive structural variation (Saxena et al., 2014), and a high proportion of repetitive sequences and transposable elements (*e.g.*, over 80% in maize) (Stitzer et al., 2021), all of which introduce ambiguity in sequence representation and increase noise in training data, ultimately degrading model performance. In addition, plant gene expression is dynamically regulated by environmental factors (Ben Rejeb et al., 2014; Greenham and McClung, 2015), including photoperiod, abiotic stresses (*e.g.*, drought, salinity, and extreme temperatures), and biotic stresses (*e.g.*, pathogen infection and pest damage). These conditions require broader model generalizability to effectively capture the complex response mechanisms they induce. Finally, the scarcity and limited diversity of plant datasets further constrain the effective use of FMs in plant molecular biology (Lam et al., 2024).

Recent advances, such as the integration of high-resolution plant omics data and innovative architectural designs, have driven the development of FMs for plant molecular biology. These models enable new approaches to genetic analysis, trait prediction, and precision breeding in plants (Lam et al., 2024). This review systematically examines the progress of biological FMs at multiple molecular levels, highlights the latest advances in FMs for plant molecular biology, discusses current research paradigms and technical bottlenecks, and proposes future directions for FM-driven research in plant science.

## Multi-level research dynamics of biological FMs

Biological FMs provide analytical frameworks that span DNA, RNA, protein, and single-cell levels. By integrating sequence, structure, and multi-omics data, they enable cross-scale investigations ranging from molecular mechanisms to system-wide processes. This progress in biological FMs has been instrumental in advancing the development of plant-specific FMs, offering critical insights into how similar frameworks can be adapted to the unique challenges of plant molecular biology.

### DNA-level FMs

The development of DNA-level FMs has transformed genomic research, from localized sequence analysis to holistic analysis of entire genomes. Early models, such as DNABERT (Ji et al., 2021), identify regulatory elements (e.g., promoters and enhancers) by employing k-mer tokenization—a method that segments DNA sequences into overlapping subsequences of length k—and the Bidirectional Encoder Representations from Transformers (BERT) architecture. DNABERT-2 (Zhou et al., 2023) improves both efficiency and accuracy through Byte Pair Encoding (BPE) and low-rank adaptation. Nucleotide Transformer (Dalla-Torre et al., 2024) also adopts the Transformer architecture and supports a 6-kb context window in its original version, with the recently released v2 extending support to 12-kb, further improving the modeling of long-range dependencies in DNA. GROVER (Sanabria et al., 2024), trained using BPE and a custom next k-mer prediction task, constructs a “genomic grammar handbook” that models human DNA sequence rules and excels in promoter identification and protein–DNA binding tasks. More recent models, HyenaDNA (Nguyen et al., 2023) and Evo (Nguyen et al., 2024), substantially enhance genome design efficiency through innovations like the Hyena operator and StripedHyena architecture, enabling the processing of sequences spanning millions of base pairs and uncovering cross-species co-evolutionary relationships. This technological trajectory reflects a journey from analyses that identify core elements to megabase-scale sequence interpretation, cross-species generalization, and ultimately genome-scale engineering. Additionally, GPN-MSA (Benegas et al., 2025) represents a distinct type of FM by incorporating multi-species alignment data to enhance the prediction of functional variants in non-coding regions.

### RNA-level FMs

RNA FMs are emerging as vital tools for unraveling the intricate relationships among RNA sequences, structures, and functions. RNABERT (Akiyama and Sakakibara, 2022) and RNA-FM (Chen et al., 2022) provide the groundwork and set foundational benchmarks in this field. Numerous models with distinct strengths have been developed. SpliceBERT (Chen et al., 2024a) and CodonBERT (Li et al., 2024b) improve splice-site prediction and codon optimization, which enhance the accuracy of gene expression analysis. DGRNA (Yuan et al., 2024) pushes the boundaries further using the bidirectional Mamba2 architecture to process long sequences, outperforming conventional models in tasks such as non-coding RNA classification and splice-site prediction. RNA-MSM (Zhang et al., 2024b), a multiple sequence alignment-based model, uses RNAcmap and an unsupervised strategy to enhance RNA structure and function prediction through evolutionary insights, whereas RiNALMo (Penić et al., 2024), pre-trained on 36 million non-coding RNAs, excels in RNA structure prediction and generalizes well to novel RNA families. LAMAR (Zhou et al., 2024) uses large-scale pre-training to decode RNA splicing and translational regulation, whereas GenerRNA (Zhao et al., 2024a), a GPT-2-like generative model, designs functional RNAs with predicted secondary structures, showing promise for synthetic biology applications. RNAGenesis (Zhang et al., 2024c) integrates a latent variable diffusion framework and demonstrates strong performance in aptamer design and CRISPR sgRNA optimization. These models illustrate the evolution of RNA FMs from initial benchmarks to specialized task performance, ultimately advancing both generative and integrative capabilities in RNA biology research.

### Protein-level FMs

Protein FMs, supported by massive training datasets, are revolutionizing structural prediction, functional analysis, and directed protein design. These models have evolved from representations of single data types to multi-modal collaborative frameworks, and are categorized as structure-guided, sequence-driven, or multi-modal fusion models. Structure-guided models focus on three-dimensional protein structures and spatial amino acid interactions. For instance, GearNet (Zhang et al., 2022) dynamically encodes residue-level geometric features using multi-relational graph convolution; SaProt (Su et al., 2023) improves function prediction by incorporating residue types and discretized structural tokens representing 3D interactions; Chroma (Ingraham et al., 2023) and RFDiffusion (Watson et al., 2023) enable precise folding control through topologically constrained diffusion processes. In contrast, sequence-driven models use large-scale evolutionary data to analyze the complex interplay between sequence, structure, and function. The ESM (Lin et al., 2022) and ProtTrans (Elnaggar et al., 2021) series capture long-range dependencies to improve function and folding predictions, whereas ProGen2 (Nijkamp et al., 2023) and ProteinBERT (Brandes et al., 2022) are pre-trained via SSL on large-scale sequence data to enhance function prediction, with ProGen2 additionally supporting protein design. Multi-modal fusion models integrate diverse data types to enhance performance: ProtST (Xu et al., 2023) and ProteinAligner (Zhang et al., 2024a) combine structural data with biomedical texts to refine function classification; AlphaFold3 (Abramson et al., 2024) extends structure prediction to complexes involving DNA/RNA and post-translational modifications; Chai-1 (team et al., 2024) supports unified structure prediction of proteins, small molecules, and DNA/RNA with a focus on drug discovery; and ESM3 (Hayes et al., 2025) enables multi-modal molecular modeling by jointly generating sequence, structure, and function. This progression highlights the increasing integration of diverse data modalities to build robust tools for protein research and applications.

### Single-cell-level FMs

Single-cell FMs are revolutionizing systems biology by bridging cellular mechanisms with tissue-level phenotypes, primarily in transcriptomic and epigenetic modeling. In transcriptomics, models have evolved from basic gene expression prediction and cell type annotation to multi-task modeling and cross-species generalization. Pioneering models such as scBERT (Yang et al., 2022) and Geneformer (Theodoris et al., 2023) use Transformer architectures for context-aware gene expression prediction and cell type classification. Subsequent models, including scGPT (Cui et al., 2024) and scFoundation (Hao et al., 2024), pre-trained on larger datasets, achieve enhanced generalizability and improved accuracy in multiple tasks. scLong (Bai et al., 2024) incorporates a Performer encoder and Gene Ontology information to improve predictions of genetic perturbation outcomes. scMulan (Bian et al., 2024) introduces a multi-task generative framework capable of simultaneously performing cell type annotation, gene expression prediction, and generation of specific cell subpopulations. GeneCompass (Yang et al., 2024) further improves cross-species generalizability by integrating large-scale single-cell datasets with prior biological knowledge, such as gene co-expression relationships. In contrast, epigenetic modeling has progressed from basic associations between chromatin accessibility and gene expression to more advanced capabilities such as detailed analysis of cell heterogeneity and cross-modal data integration. EpiAgent (Chen et al., 2024b) demonstrates strong performance in perturbation response and noise-resistant annotation by emphasizing cell heterogeneity modeling. GET (Fu et al., 2025) accurately predicts gene expression across cell types using chromatin accessibility and gene sequence data, whereas EpiFoundation (Wu et al., 2025a) improves gene activity prediction through supervised learning across multiple data types. Collectively, single-cell FMs are enabling deeper data integration, finer characterization of cell heterogeneity, and more efficient cross-modal processing, thereby expanding our understanding of complex biological systems.

Biological FMs have also shown significant potential in a wide range of additional tasks, including methylation prediction [e.g., MethylGPT (Ying et al., 2024), CpGPT (de Lima Camillo et al., 2024)] and antibody design [e.g., SyntheMol (Swanson et al., 2024)]. These advances highlight the transformative potential of FMs in biological research. However, their application in plant sciences remains underexplored, particularly in areas other than DNA and RNA analysis. For comprehensive updates on biological FMs, readers are referred to recent reviews (Li et al., 2024a; Guo et al., 2025b; Khan et al., 2025). The following sections delve into the current state and future prospects of plant-specific FMs, emphasizing their potentially critical role in addressing unique challenges in plant molecular biology and beyond (**Table 1**).

**Table 1:** Specific and universal FMs for plant molecular biology.

Model name	Model type	Model architecture	Pre-training strategy	Training data	Task types	Computing resources used for training	Parameter sizes	Resource consumption level of inference	Innovations
GPN	Plant DNA model	CNN	MLM	Reference genome assemblies for *Arabidopsis* and seven Brassicaceae species	Genomic functional element identification, variant effect prediction, *etc.*	Four days with four NVIDIA A100–80 GB GPUs	65 million	Medium	- The first DNA language model for plants, using CNN to learn genomic sequences - Optimized loss weights improve prediction for non-repetitive regions - Unsupervised zero-shot variant effect prediction; excellent at rare variant identification
AgroNT	Plant DNA model	Transformer	MLM	10.5 million genomic sequences across 48 edible plant species	Eight tasks, including polyadenylation site prediction, splice-site prediction, chromatin accessibility prediction, *etc.*	Google TPU-V4–1024 machine containing 512 devices	1 billion	High	- The first Transformer pre-training model focused on edible plants - Assesses mutation effects and enables variant prediction via simulated mutagenesis - Constructs PGB evaluation datasets
PDLLMs	Plant DNA model	Hybrid architecture with tokenization strategies	MLM, CLM	22 reference genomes from 14 kinds of plant species	Nine tasks, including promoter prediction, chromatin accessibility recognition, cross-species lncRNA prediction, *etc.*	One NVIDIA RTX4090 GPUs	89 million ~ 152 million	Medium	- Enables efficient training and inference on consumer-grade GPUs - Fine-tuned 198 specialized models for nine downstream tasks - Reduces technical barriers by providing open-source code, pre-trained weights, and a web platform
PlantCaduceus	Plant DNA model	Caduceus, Mamba	MLM	Genomes from 16 angiosperm species	Cross-species genomic element prediction, deleterious mutation identification, evolutionary conservation analysis, *etc.*	Not mentioned	20 million ~ 225 million	Low, Medium	- Single-nucleotide bidirectional context modeling based on Caduceus and Mamba architectures - Enhances cross-species prediction of translation initiation and termination sites and deleterious mutations - Improvements in parameter efficiency
PlantRNA-FM	Plant RNA model	Transformer	MLM	25 million RNA sequences, annotations, and structural prediction data from 1,124 plant species	RNA secondary structure prediction, gene region annotation, translation efficiency prediction, *etc.*	Over three weeks on four NVIDIA A100 GPUs	35 million	Low	- The first plant RNA interpretable FM combining RNA sequences, structures, and functions - Learned to understand the grammar and regulatory logic of RNA sequences and structures - Systematically reveals structural principles and positional effects of translation-related RNA motifs
GENERator	Universal DNA model	Transformer	NTP	386 billion nucleotides from various eukaryotes	Genomic element prediction, protein family DNA sequence design, enhancer design, *etc.*	368 hours on 32 NVIDIA A100 GPUs	1.2 billion	High	- Performs long-context modeling by integrating multi-scale biological sequences - Uses a “gene sequence training” scheme - Generates protein-coding sequences and performs regulatory element engineering
Evo 2	Universal DNA model	StripedHyena 2	NTP	Over 9.3 trillion nucleotides spanning all domains of life, including archaea, prokaryotes, fungi, protists, plants, and animals	Genome design, mutation pathogenicity prediction, gene expression prediction, non-coding DNA functional modeling, *etc.*	Not mentioned	1 billion, 7 billion, 40 billion	High, Extremely High	- The largest biological FM to date - Models a one million-nucleotide context window at single-nucleotide resolution - Models co-evolutionary relationships between coding and non-coding sequences - Supports feature interpretation from molecular to genomic scales - Comprehensively predicts and generates sequences across all domains of life

MLM, Masked Language Model; CLM, Causal Language Modelling; NTP, Next-token Prediction; CNN, Convolutional Neural Network; PGB, Plant Genomic Benchmark; FM, Foundation Model. The classification of inference resource consumption level is based on model parameter size: Low (0–50 million), Medium (50 million–500 million), High (500 million–5 billion), and Extremely High (>5 billion).

## FMs designed for plant molecular biology

### Genomic pre-trained network (GPN)

GPN (Benegas et al., 2023), the first DNA LLM specifically tailored for plants, incorporates CNNs and a masked language model (MLM) pre-training strategy to model functional constraints and sequence patterns, using training data from *Arabidopsis* and seven other Brassicaceae genomes. By adjusting the loss weights for repetitive regions, it improves prediction accuracy in non-repetitive functional regions such as coding sequences (96% accuracy), introns, and non-coding RNAs, outperforming traditional k-mer-based methods. GPN excels in variant effect prediction, with its score showing a stronger correlation with rare alleles than phyloP and phastCons (Pollard et al., 2010), which rely on whole-genome alignments of 18 closely related species. Notably, it enables unsupervised cross-species promoter motif identification without requiring functional genomics data or multi-species alignment. GPN provides an efficient, unsupervised approach to variant effect prediction, enabling more precise fine mapping of genome-wide association study loci and multi-gene risk assessment in plants.

### Agronomic nucleotide transformer (ArgoNT)

AgroNT (Mendoza-Revilla et al., 2024) is pre-trained on 10.5 million genomic sequences from 48 edible plant species through MLM using fixed-size k-mer tokenization and a parameter-efficient fine-tuning method (IA3). By replacing the model’s head layer, AgroNT enables zero-shot transfer learning across various tasks, delivering robust performance in downstream applications such as regulatory feature identification and gene expression prediction. It also supports mutation effect evaluation and variant characterization through large-scale simulated saturation mutagenesis. In addition, AgroNT introduces the Plant Genomic Benchmark (PGB), a plant-specific dataset designed to assess performance across eight tasks, offering standardized criteria for model evaluation in plant genomics.

### Plant DNA large language models (PDLLMs)

PDLLMs (Liu et al., 2025) are optimized DNA LLMs designed to address the challenges of plant genomic analysis. They are built on a combination of multiple foundational model architectures (*i.e.*, Mamba, BERT, GPT, Gemma, and Nucleotide Transformer) and tokenization strategies (*i.e.*, single nucleotide, k-mer, and BPE), and pre-trained using either MLM or causal language modelling (CLM) strategies. Key strengths include: 1) enhanced detection of plant-specific regulatory elements, enabled by pre-training on 14 plant genomes; 2) broad adaptability, achieved through fine-tuning on 10 additional plant datasets to create 198 models specialized for nine downstream tasks, such as predicting core promoters and sequence conservation; 3) a lightweight design suitable for training and inference on consumer-grade GPUs or CPUs, eliminating dependence on high-performance hardware. In addition, PDLLMs are released with pre-training codes and supported by a web-based platform, offering an accessible and scalable tool for plant genomics and precision breeding, especially in resource-constrained environments.

### PlantCaduceus

PlantCaduceus (Zhai et al., 2024), built on the Caduceus (Schiff et al., 2024) and Mamba architectures, incorporates a reverse-complement-equivariant architecture to account for the double-stranded nature of DNA. It addresses the high prevalence of repetitive elements in plant genomes through optimized pre-processing strategies, such as down-weighting repetitive elements and balancing sampling from non-coding regions. Pre-trained with MLM on 16 angiosperm genomes and fine-tuned with a minimal amount of labeled *Arabidopsis* data, PlantCaduceus demonstrated high transferability by accurately predicting translation initiation and termination sites, as well as splice sites, in maize. It also predicts evolutionary conservation and generalizes across species from single-sequence inputs, outperforming traditional supervised models and delivering zero-shot variant effect prediction with triple the sensitivity of PhyloP (Pollard et al., 2010). Compared with baseline models like GPN (Benegas et al., 2023) and AgroNT (Mendoza-Revilla et al., 2024), PlantCaduceus demonstrates superior cross-species performance with greater parameter efficiency.

### PlantRNA-FM

PlantRNA-FM (Yu et al., 2024), the first RNA FM tailored for plants, offers notable advantages in multi-modal RNA data processing and functional interpretability. Pre-trained using MLM on a dataset comprising 25 million RNA sequences (totaling 54 billion nucleotides), annotations, and structural data from 1,124 plant species, it captures the extensive diversity of plant transcriptome landscapes. The model uses single-nucleotide resolution tokenization and rotary position embedding, ensuring that RNA structural motifs are learned as unified elements and reducing the embedding layer parameters by 30%. In tasks such as gene region annotation and translation efficiency prediction, PlantRNA-FM achieves substantial improvements in accuracy. Furthermore, through attention contrast matrices and unsupervised hierarchical clustering, it can identify functional RNA motifs, providing interpretable insights into plant RNA regulatory networks.

## Universal FMs applicable to plants

### GENERator

GENERator (Wu et al., 2025b) is a generative genomic FM built on a Transformer decoder architecture, featuring 1.2 billion parameters and a context window of up to 98,000 bases in length. Pre-trained using a next-token prediction (NTP) strategy on a cross-species eukaryotic DNA dataset containing 386 billion nucleotides, including over 7 million genes and 30 billion bases from plants, GENERator exhibits excellent cross-species generalizability. Its key innovation, the “gene sequence training” scheme, prioritizes functional gene regions and uses a 6-mer tokenizer to optimize DNA sequence modeling, filtering out evolutionarily redundant sequences to identify functional regions. Despite its modest parameter size, GENERator can design coding sequences structurally aligned with target protein families or create synthetic promoters with user-specified activity levels, making it a valuable tool for plant synthetic biology and genetic engineering.

### Evo 2

Evo 2 (Brixi et al., 2025) represents a recent breakthrough in multi-modal FMs, with up to 40 billion parameters pre-trained through NTP on over 9.3 trillion nucleotides from species across all domains of life. It uses the innovative StripedHyena2 architecture (Ku et al., 2025), which combines convolutional and attention mechanisms to process sequences up to one million bases long, effectively capturing long-range regulatory dependencies within 3D genomic structures. While designed as a general-purpose genomic FM, Evo 2 is extensively trained on plant genomic data, enabling effective performance on plant-specific tasks, such as rapid prediction of gene mutation effects to support disease resistance breeding and genetic research. It also facilitates plant-specific DNA sequence design and complex systems engineering, driving advances in plant synthetic biology and biotechnology.

## Opportunities and challenges in plant FM construction

Currently, FMs in plant molecular biology primarily focus on DNA and RNA levels. Notably, there is a significant gap in the development of FMs for other critical areas of plant biology, such as protein structure and function, single-cell dynamics, and epigenetic regulation. Although general protein FMs (like the ESM series) can be adapted for plant research, the complexity of plant-specific environmental response mechanisms underscores the need for specialized protein FMs. In the single-cell field, the limited amount of training data is gradually being addressed by resources such as the scPlantDB database (He et al., 2023), which includes 2.5 million cells from 17 plant species, offering promising opportunities for the development of plant-specific single-cell FMs. However, the development of FMs for plant molecular biology continues to face significant challenges.

### Data heterogeneity and annotation bottlenecks

Plant genomes exhibit remarkable diversity, with pronounced differences across species in gene structures, regulatory elements, and the functions of non-coding regions. For instance, promoters may follow different patterns between monocots and dicots (Kumari and Ware, 2013), necessitating training datasets that include representative genomes from a broad phylogenetic spectrum. However, high-quality annotated data are costly to obtain, especially for experimental datasets related to epigenetic modifications (*e.g.*, H3K4me3, H3K27ac) (Holder et al., 2017) or the functional validation of non-coding RNAs (Xu et al., 2021), which limits model generalizability.

### Biological adaptability of model architectures

Current LLM architectures, such as BERT and GPT, were primarily designed for human NLP and face semantic mismatch challenges when directly applied to biological sequences. The syntactic rules of DNA sequences, such as codon reading frames and splice sites, differ fundamentally from linguistic syntax (Sanabria et al., 2024; Theodoris, 2024), requiring strategies such as tailored tokenization (e.g., k-mer and BPE) or specialized pre-training. Research shows that single-nucleotide tokenization performs better in regression tasks like promoter strength prediction, whereas 6-mer tokenization is more effective for the classification of regulatory elements (Liu et al., 2025), highlighting the importance of task-specific architectural optimization.

### Cross-species generalizability and functional interpretability

Plant FMs need to accurately support knowledge transfer across species despite genomic differences. For instance, PDLLMs exhibit different performance in histone modification prediction between maize and *Arabidopsis* (Liu et al., 2025), underscoring the need to integrate evolutionary context to improve generalizability. In addition, the black-box nature of current models complicates biological interpretation (Shen and Li, 2024), making the development of methods to elucidate model decision-making logic an ongoing challenge.

### Computational efficiency and resource limitations

Architectures like Mamba reduce computational complexity using state space models (*e.g.*, the PDLLM Plant-DNAMamba outperforms AgroNT in multiple tasks with only 130 million parameters (Liu et al., 2025)); however, processing long sequences from plant genomes remains a challenge due to the high proportion of repetitive sequences and widely dispersed regulatory elements (Zhai et al., 2024). In addition, many plant research laboratories lack access to high-performance computing resources, necessitating models optimized for consumer-grade GPUs through improved architectural design and parameter compression.

## Future prospects for plant molecular biology FMs

Future research on plant molecular biology FMs should prioritize the optimization of existing models and the exploration of new frontiers in emerging fields, with emphasis on three main areas: model generalizability, multi-modal integration and interpretability, and computational efficiency and sustainability.

### Model generalizability

Improving FM generalizability is essential to unlocking their practical potential. Current researches often focus on model plant species, but major agricultural crops like maize and wheat, characterized by complex polyploidy and high repetitive content (Garg et al., 2024), demand greater cross-species adaptability. Future strategies could include multi-species joint training to accommodate genomic diversity, and dynamic architectures such as Mixture of Experts (MoE) (Shazeer et al., 2017) may enable sub-models to be tailored to specific crop traits. In addition, using few-shot or zero-shot learning alongside SSL (*e.g.*, contrastive learning) could facilitate functional element identification in non-model crops (Zhao et al., 2024b), reducing dependence on large datasets.

### Multi-modal integration and interpretability

Multi-modal integration and interpretability are vital for addressing the complexity of biological systems. Current models, often limited to single-modality inputs (Lam et al., 2024), underscore the need to incorporate multi-omics data in plant biology. Evo 2, for instance, successfully models and learns the information flow and encoding rules of the central dogma of molecular biology through cross-species multi-modal integration (Brixi et al., 2025), which could be further enhanced with data from plant imaging and dynamic growth sensors to predict plant development and environmental responses. To address the black-box nature of increasingly sophisticated models, interpretability techniques such as attention visualization and feature attribution could help connect decision logic to biological patterns (Huang et al., 2025). Future plant molecular FMs could also integrate causal inference methods to identify key gene regulatory network nodes, thereby providing interpretable biological insights to inform experimental design.

### Computational optimization and sustainability

Optimizing computational efficiency is essential for the large-scale deployment of FMs. Although large-scale models like Evo 2 and ESM3 offer excellent performance, their massive parameter sizes impose high training and inference costs, limiting their broader adoption in plant science. Techniques such as model compression (*e.g.*, distillation, quantization, and pruning) can reduce resource demands without substantially compromising performance. For instance, the DeepSeek team distilled a 671B-parameter model into a 7B-parameter version for faster and cheaper inference (Guo et al., 2025a), and PDLLMs use an optimized architecture for efficient inference on consumer-grade GPUs (Liu et al., 2025). Future plant molecular biology FMs could leverage these techniques, along with hardware acceleration (*e.g.*, TensorRT) and dynamic resource allocation, to overcome technical barriers in plant research. Furthermore, advances in federated learning could support distributed training across institutions (Li et al., 2020), preserving data privacy while enhancing generalizability and promoting global collaboration in plant science and sustainable agriculture.

In summary, plant molecular biology FMs serve as a bridge between artificial intelligence and plant science, offering powerful tools to decode the intricacies of plant biology and paving the way for smarter and more sustainable agriculture. As technological capabilities advance, FMs are poised to assume a pivotal role in shaping the future of plant and agricultural research, empowering humanity to confront the ever-growing global climate challenges.

## References

[B1] Abramson J. Adler J. Dunger J. Evans R. Green T. Pritzel A.. (2024). Accurate structure prediction of biomolecular interactions with AlphaFold 3. Nature 630, 493–500. doi: 10.1038/s41586-024-07487-w 38718835 PMC11168924

[B2] Akiyama M. Sakakibara Y. (2022). Informative RNA base embedding for RNA structural alignment and clustering by deep representation learning. NAR Genomics Bioinf. 4, lqac012. doi: 10.1093/nargab/lqac012 PMC8862729 35211670

[B3] Bai D. Mo S. Zhang R. Luo Y. Gao J. Yang J. P.. (2024). scLong: A billion-parameter foundation model for capturing long-range gene context in single-cell transcriptomics. bioRxiv. doi: 10.1101/2024.11.09.622759 PMC12982784 41639087

[B4] Benegas G. Albors C. Aw A. J. Ye C. Song Y. S. (2025). A DNA language model based on multispecies alignment predicts the effects of genome-wide variants. Nat. Biotechnol., 1–6. doi: 10.1038/s41587-024-02511-w 39747647

[B5] Benegas G. Batra S. S. Song Y. S. (2023). DNA language models are powerful predictors of genome-wide variant effects. Proc. Natl. Acad. Sci. 120, e2311219120. doi: 10.1073/pnas.2311219120 37883436 PMC10622914

[B6] Ben Rejeb I. Pastor V. Mauch-Mani B. (2014). Plant responses to simultaneous biotic and abiotic stress: molecular mechanisms. Plants 3, 458–475. doi: 10.3390/plants3040458 27135514 PMC4844285

[B7] Bian H. Chen Y. Dong X. Li C. Hao M. Chen S. (2024). “scMulan: a multitask generative pre-trained language model for single-cell analysis,” in International Conference on Research in Computational Molecular Biology (Springer, Cham, Switzerland: Springer), 479–482. doi: 10.1007/978-1-0716-3989-4_57

[B8] Brandes N. Ofer D. Peleg Y. Rappoport N. Linial M. (2022). ProteinBERT: a universal deep-learning model of protein sequence and function. Bioinformatics 38, 2102–2110. doi: 10.1093/bioinformatics/btac020 35020807 PMC9386727

[B9] Brixi G. Durrant M. G. Ku J. Poli M. Brockman G. Chang D.. (2025). Genome modeling and design across all domains of life with Evo 2. bioRxiv. doi: 10.1101/2025.02.18.638918 PMC13128491 41781614

[B10] Chen J. Hu Z. Sun S. Tan Q. Wang Y. Yu Q.. (2022). Interpretable RNA foundation model from unannotated data for highly accurate RNA structure and function predictions. arXiv preprint arXiv:2204.00300. doi: 10.48550/arXiv.2204.00300

[B11] Chen X. Li K. Cui X. Wang Z. Jiang Q. Lin J.. (2024b). EpiAgent: Foundation model for single-cell epigenomic data. bioRxiv. doi: 10.1101/2024.12.19.629312 40999099

[B12] Chen K. Zhou Y. Ding M. Wang Y. Ren Z. Yang Y. (2024a). Self-supervised learning on millions of primary RNA sequences from 72 vertebrates improves sequence-based RNA splicing prediction. Briefings Bioinf. 25, bbae163. doi: 10.1093/bib/bbae163 PMC11009468 38605640

[B13] Cui H. Wang C. Maan H. Pang K. Luo F. Duan N.. (2024). scGPT: toward building a foundation model for single-cell multi-omics using generative AI. Nat. Methods 21, 1470–1480. doi: 10.1038/s41592-024-02201-0 38409223

[B14] Dalla-Torre H. Gonzalez L. Mendoza-Revilla J. Lopez Carranza N. Grzywaczewski A. H. Oteri F.. (2024). Nucleotide Transformer: building and evaluating robust foundation models for human genomics. Nat. Methods 22, 287–297. doi: 10.103a/5415924024-02523-z 39609566 PMC11810778

[B15] de Lima Camillo L. P. Sehgal R. Armstrong J. Higgins-Chen A. T. Horvath S. Wang B. (2024). CpGPT: a foundation model for DNA methylation. bioRxiv. doi: 10.1101/2024.10.24.619766

[B16] Devlin J. Chang M.-W. Lee K. Toutanova K. (2019). “Bert: Pre-training of deep bidirectional transformers for language understanding,” in Proceedings of the 2019 conference of the North American chapter of the association for computational linguistics: human language technologies, vol. 1. (Minneapolis, MN, USA: Association for Computational Linguistics), 4171–4186. doi: 10.18653/v1/N19-1423

[B17] Elnaggar A. Heinzinger M. Dallago C. Rehawi G. Wang Y. Jones L.. (2021). Prottrans: Toward understanding the language of life through self-supervised learning. IEEE Trans. Pattern Anal. Mach. Intell. 44, 7112–7127. doi: 10.1109/TPAMI.2021.3095381 34232869

[B18] Fu X. Mo S. Buendia A. Laurent A. P. Shao A. Alvarez-Torres M.. (2025). A foundation model of transcription across human cell types. Nature 637, 965–973. doi: 10.1038/s41586-024-08391-z 39779852 PMC11754112

[B19] Garg V. Bohra A. Mascher M. Spannagl M. Xu X. Bevan M. W.. (2024). Unlocking plant genetics with telomere-to-telomere genome assemblies. Nat. Genet. 56, 1788–1799. doi: 10.1038/s41588-024-01830-7 39048791

[B20] Greenham K. McClung C. R. (2015). Integrating circadian dynamics with physiological processes in plants. Nat. Rev. Genet. 16, 598–610. doi: 10.1038/nrg3976 26370901

[B21] Guo F. Guan R. Li Y. Liu Q. Wang X. Yang C.. (2025b). Foundation models in bioinformatics. Natl. Sci. Rev. 12, nwaf028. doi: 10.1093/nsr/nwaf028 40078374 PMC11900445

[B22] Guo D. Yang D. Zhang H. Song J. Zhang R. Xu R.. (2025a). Deepseek-r1: Incentivizing reasoning capability in llms via reinforcement learning. arXiv preprint arXiv:2501.12948. doi: 10.48550/arXiv.2501.12948

[B23] Hao M. Gong J. Zeng X. Liu C. Guo Y. Cheng X.. (2024). Large-scale foundation model on single-cell transcriptomics. Nat. Methods 21, 1481–1491. doi: 10.1038/s41592-024-02305-7 38844628

[B24] Hayes T. Rao R. Akin H. Sofroniew N. J. Oktay D. Lin Z.. (2025). Simulating 500 million years of evolution with a language model. Science 387, eads0018. doi: 10.1126/science.ads0018 39818825

[B25] He Z. Luo Y. Zhou X. Zhu T. Lan Y. Chen D. (2023). scPlantDB: a comprehensive database for exploring cell types and markers of plant cell atlases. Nucleic Acids Res. 52, D1629–D1638. doi: 10.1093/nar/gkad706 PMC10767885 37638765

[B26] Holder L. B. Haque M. M. Skinner M. K. (2017). Machine learning for epigenetics and future medical applications. Epigenetics 12, 505–514. doi: 10.1080/15592294.2017.1329068 28524769 PMC5687335

[B27] Hou X. Zhao Y. Liu Y. Yang Z. Wang K. Li L.. (2024). Large language models for software engineering: A systematic literature review. ACM Trans. Software Eng. Method. 33, 1–79. doi: 10.1145/3695988

[B28] Huang Y. Gomaa A. Höfler D. Schubert P. Gaipl U. Frey B.. (2025). Principles of artificial intelligence in radiooncology. Strahlentherapie und Onkologie 201, 210–235. doi: 10.1007/s00066-024-02272-0 39105746 PMC11839771

[B29] Ingraham J. B. Baranov M. Costello Z. Barber K. W. Wang W. Ismail A.. (2023). Illuminating protein space with a programmable generative model. Nature 623, 1070–1078. doi: 10.1038/s41586-023-06728-8 37968394 PMC10686827

[B30] Ji Y. Zhou Z. Liu H. Davuluri R. V. (2021). DNABERT: pre-trained Bidirectional Encoder Representations from Transformers model for DNA-language in genome. Bioinformatics 37, 2112–2120. doi: 10.1093/bioinformatics/btab083 33538820 PMC11025658

[B31] Khan W. Leem S. See K. B. Wong J. K. Zhang S. Fang R. (2025). A comprehensive survey of foundation models in medicine. IEEE Rev. Biomed. Engineering. doi: 10.1109/RBME.2025.3531360 40031197

[B32] Ku J. Nguyen E. Romero D. W. Brixi G. Yang B. Vorontsov A.. (2025). Systems and algorithms for convolutional multi-hybrid language models at scale. arXiv preprint arXiv:2503.01868. doi: 10.48550/arXiv.2503.01868

[B33] Kumari S. Ware D. (2013). Genome-wide computational prediction and analysis of core promoter elements across plant monocots and dicots. PloS One 8, e79011. doi: 10.1371/journal.pone.0079011 24205361 PMC3812177

[B34] Lam H. Y. I. Ong X. E. Mutwil M. (2024). Large language models in plant biology. Trends Plant Science 29, 1145–11155. doi: 10.1016/j.tplants.2024.04.013 38797656

[B35] Li L. Fan Y. Tse M. Lin K.-Y. (2020). A review of applications in federated learning. Comput. Ind. Eng. 149, 106854. doi: 10.1016/j.cie.2020.106854

[B36] Li Q. Hu Z. Wang Y. Li L. Fan Y. King I.. (2024a). Progress and opportunities of foundation models in bioinformatics. Briefings Bioinf. 25, bbae548. doi: 10.1093/bib/bbae548 PMC11512649 39461902

[B37] Li S. Moayedpour S. Li R. Bailey M. Riahi S. Kogler-Anele L.. (2024b). CodonBERT large language model for mRNA vaccines. Genome Res. 34, 1027–1035. doi: 10.1101/gr.278870.123 38951026 PMC11368176

[B38] Lin Z. Akin H. Rao R. Hie B. Zhu Z. Lu W.. (2022). Evolutionary-scale prediction of atomic-level protein structure with a language model. Science 379, 1123–1130. doi: 10.1126/science.ade2574 36927031

[B39] Liu G. Chen L. Wu Y. Han Y. Bao Y. Zhang T. (2025). PDLLMs: A group of tailored DNA large language models for analyzing plant genomes. Mol. Plant 18, 175–178. doi: 10.1016/j.molp.2024.12.006 39659015

[B40] Liu J. Yang M. Yu Y. Xu H. Li K. Zhou X. (2024). Advancing bioinformatics with large language models: components, applications and perspectives. arXiv preprint arXiv:2401.04155. doi: 10.48550/arXiv.2503.01868 PMC13354062 42430786

[B41] Mendoza-Revilla J. Trop E. Gonzalez L. Roller M. Dalla-Torre H. de Almeida B. P.. (2024). A foundational large language model for edible plant genomes. Commun. Biol. 7, 835. doi: 10.1038/s42003-024-06465-2 38982288 PMC11233511

[B42] Nguyen E. Poli M. Durrant M. G. Kang B. Katrekar D. Li D. B.. (2024). Sequence modeling and design from molecular to genome scale with Evo. Science 386, eado9336. doi: 10.1126/science.ado9336 39541441 PMC12057570

[B43] Nguyen E. Poli M. Faizi M. Thomas A. Wornow M. Birch-Sykes C.. (2023). Hyenadna: Long-range genomic sequence modeling at single nucleotide resolution. Adv. Neural Inf. Process. Syst. 36, 43177–43201. doi: 10.5555/3666122.3667994

[B44] Nijkamp E. Ruffolo J. A. Weinstein E. N. Naik N. Madani A. (2023). Progen2: exploring the boundaries of protein language models. Cell Syst. 14, 968–978.e963. doi: 10.1016/j.cels.2023.10.002 37909046

[B45] Penić R. J. Vlašić T. Huber R. G. Wan Y. Šikić M. (2024). Rinalmo: General-purpose rna language models can generalize well on structure prediction tasks. arXiv preprint arXiv:2403.00043. doi: 10.48550/arXiv.2403.00043 PMC12219582 40593636

[B46] Pollard K. S. Hubisz M. J. Rosenbloom K. R. Siepel A. (2010). Detection of nonneutral substitution rates on mammalian phylogenies. Genome Res. 20, 110–121. doi: 10.1101/gr.097857.109 19858363 PMC2798823

[B47] Radford A. Narasimhan K. Salimans T. Sutskever I. (2018). Improving language understanding by generative pre-training.

[B48] Sanabria M. Hirsch J. Joubert P. M. Poetsch A. R. (2024). DNA language model GROVER learns sequence context in the human genome. Nat. Mach. Intell. 6, 911–923. doi: 10.1038/s42256-024-00872-0

[B49] Saxena R. K. Edwards D. Varshney R. K. (2014). Structural variations in plant genomes. Briefings Funct. Genomics 13, 296–307. doi: 10.1093/bfgp/elu016 PMC4110416 24907366

[B50] Schiff Y. Kao C.-H. Gokaslan A. Dao T. Gu A. Kuleshov V. (2024). Caduceus: Bi-directional equivariant long-range dna sequence modeling. arXiv preprint arXiv:2403.03234. doi: 10.48550/arXiv.2403.03234 PMC12189541 40567809

[B51] Shazeer N. Mirhoseini A. Maziarz K. Davis A. Le Q. Hinton G.. (2017). Outrageously large neural networks: The sparsely-gated mixture-of-experts layer. arXiv preprint arXiv:1701.06538. doi: 10.48550/arXiv.1701.06538

[B52] Shen X. Li X. (2024). Deep-learning methods for unveiling large-scale single-cell transcriptomes. Cancer Biol. Med. 20, 972. doi: 10.20892/j.issn.2095-3941.2023.0436 38318925 PMC10845931

[B53] Stitzer M. C. Anderson S. N. Springer N. M. Ross-Ibarra J. (2021). The genomic ecosystem of transposable elements in maize. PloS Genet. 17, e1009768. doi: 10.1371/journal.pgen.1009768 34648488 PMC8547701

[B54] Su J. Han C. Zhou Y. Shan J. Zhou X. Yuan F. (2023). Saprot: Protein language modeling with structure-aware vocabulary. bioRxiv. doi: 10.1101/2023.10.01.56034

[B55] Swanson K. Liu G. Catacutan D. B. Arnold A. Zou J. Stokes J. M. (2024). Generative AI for designing and validating easily synthesizable and structurally novel antibiotics. Nat. Mach. Intell. 6, 338–353. doi: 10.1038/s42256-024-00809-7

[B56] team C. D. Boitreaud J. Dent J. McPartlon M. Meier J. Reis V.. (2024). Chai-1: Decoding the molecular interactions of life. BioRxiv. doi: 10.1101/2024.10.10.615955

[B57] Theodoris C. V. (2024). Learning the language of DNA. Science 386, 729–730. doi: 10.1126/science.adt3007 39541478

[B58] Theodoris C. V. Xiao L. Chopra A. Chaffin M. D. Al Sayed Z. R. Hill M. C.. (2023). Transfer learning enables predictions in network biology. Nature 618, 616–624. doi: 10.1038/s41586-023-06139-9 37258680 PMC10949956

[B59] Vaswani A. Shazeer N. Parmar N. Uszkoreit J. Jones L. Gomez A. N.. (2017). Attention is all you need. Adv. Neural Inf. Process. Syst. 30, 6000–6010. doi: 10.5555/3295222.3295349

[B60] Walkowiak S. Gao L. Monat C. Haberer G. Kassa M. T. Brinton J.. (2020). Multiple wheat genomes reveal global variation in modern breeding. Nature 588, 277–283. doi: 10.1038/s41586-020-2961-x 33239791 PMC7759465

[B61] Watson J. L. Juergens D. Bennett N. R. Trippe B. L. Yim J. Eisenach H. E.. (2023). *De novo* design of protein structure and function with RFdiffusion. Nature 620, 1089–1100. doi: 10.1038/s41586-023-06415-8 37433327 PMC10468394

[B62] Wu W. Li Q. Li M. Fu K. Feng F. Ye J.. (2025b). GENERator: A long-context generative genomic foundation model. arXiv preprint arXiv:2502.07272. doi: 10.48550/arXiv.2502.07272

[B63] Wu J. Wan C. Ji Z. Zhou Y. Hou W. (2025a). EpiFoundation: A foundation model for single-cell ATAC-seq via peak-to-gene alignment. bioRxiv. doi: 10.1101/2025.02.05.636688

[B64] Xu X. Liu S. Yang Z. Zhao X. Deng Y. Zhang G.. (2021). A systematic review of computational methods for predicting long noncoding RNAs. Briefings Funct. Genomics 20, 162–173. doi: 10.1093/bfgp/elab016 33754153

[B65] Xu M. Yuan X. Miret S. Tang J. (2023). “Protst: Multi-modality learning of protein sequences and biomedical texts,” in International Conference on Machine Learning (Brooklyn, NY, USA: PMLR), 38749–38767. doi: 10.5555/3618408.3620023

[B66] Yang X. Liu G. Feng G. Bu D. Wang P. Jiang J.. (2024). GeneCompass: deciphering universal gene regulatory mechanisms with a knowledge-informed cross-species foundation model. Cell Res. 34, 830–845. doi: 10.1038/s41422-024-01034-y 39375485 PMC11615217

[B67] Yang F. Wang W. Wang F. Fang Y. Tang D. Huang J.. (2022). scBERT as a large-scale pretrained deep language model for cell type annotation of single-cell RNA-seq data. Nat. Mach. Intell. 4, 852–866. doi: 10.1038/s42256-022-00534-z

[B68] Ying K. Song J. Cui H. Zhang Y. Li S. Chen X.. (2024). MethylGPT: a foundation model for the DNA methylome. bioRxiv. doi: 10.1101/2024.10.30.621013

[B69] Yu H. Yang H. Sun W. Yan Z. Yang X. Zhang H.. (2024). An interpretable RNA foundation model for exploring functional RNA motifs in plants. Nat. Mach. Intell. 6, 1616–1625. doi: 10.1038/s42256-024-00946-z 39703563 PMC11652376

[B70] Yuan Y. Chen Q. Pan X. (2024). DGRNA: a long-context RNA foundation model with bidirectional attention Mamba2. bioRxiv. doi: 10.1101/2024.10.31.621427

[B71] Zhai J. Gokaslan A. Schiff Y. Berthel A. Liu Z.-Y. Lai W.-Y.. (2024). Cross-species modeling of plant genomes at single nucleotide resolution using a pre-trained DNA language model. bioRxiv. doi: 10.1101/2024.06.04.596709 PMC12184517 40489624

[B72] Zhang Z. Chao L. Jin R. Zhang Y. Zhou G. Yang Y.. (2024c). RNAGenesis: foundation model for enhanced RNA sequence generation and structural insights. bioRxiv. doi: 10.1101/2024.12.30.630826

[B73] Zhang Q. Ding K. Lv T. Wang X. Yin Q. Zhang Y.. (2025). Scientific large language models: A survey on biological & chemical domains. ACM Computing Surveys 57, 1–38. doi: 10.1145/3729419

[B74] Zhang L. Guo H. Schaffer L. Ko Y. S. Singh D. Rahmani H.. (2024a). ProteinAligner: A multi-modal pretraining framework for protein foundation models. bioRxiv. doi: 10.1101/2024.10.06.616870

[B75] Zhang Y. Lang M. Jiang J. Gao Z. Xu F. Litfin T.. (2024b). Multiple sequence alignment-based RNA language model and its application to structural inference. Nucleic Acids Res. 52, e3–e3. doi: 10.1093/nar/gkad1031 37941140 PMC10783488

[B76] Zhang Z. Xu M. Jamasb A. Chenthamarakshan V. Lozano A. Das P.. (2022). Protein representation learning by geometric structure pretraining. arXiv preprint arXiv:2203.06125. doi: 10.48550/arXiv.2203.06125

[B77] Zhao Z. Alzubaidi L. Zhang J. Duan Y. Gu Y. (2024b). A comparison review of transfer learning and self-supervised learning: Definitions, applications, advantages and limitations. Expert Syst. Appl. 242, 122807. doi: 10.1016/j.eswa.2023.122807

[B78] Zhao Y. Oono K. Takizawa H. Kotera M. (2024a). GenerRNA: A generative pre-trained language model for *de novo* RNA design. PLoS One 19, e0310814. doi: 10.1371/journal.pone.0310814 39352899 PMC11444397

[B79] Zhou H. Hu Y. Zheng Y. Li J. Peng J. Hu J.. (2024). A foundation language model to decipher diverse regulation of RNAs. bioRxiv. doi: 10.1101/2024.10.12.617732 PMC12462214 40993693

[B80] Zhou Z. Ji Y. Li W. Dutta P. Davuluri R. Liu H. (2023). Dnabert-2: Efficient foundation model and benchmark for multi-species genome. arXiv preprint arXiv:2306.15006. doi: 10.48550/arXiv.2306.15006

